# Comparative transcriptional and co-expression network analysis of two upland cotton accessions with extreme phenotypic differences reveals molecular mechanisms of fiber development

**DOI:** 10.3389/fpls.2023.1189490

**Published:** 2023-08-31

**Authors:** Jiasen He, Zhongyang Xu, Muhammad Tehseen Azhar, Zhen Zhang, Pengtao Li, Juwu Gong, Xiao Jiang, Senmiao Fan, Qun Ge, Youlu Yuan, Haihong Shang

**Affiliations:** ^1^ National Key Laboratory of Cotton Bio-breeding and Integrated Utilization, Zhengzhou University, Zhengzhou Henan, China; ^2^ National Key Laboratory of Cotton Bio-breeding and Integrated Utilization, Institute of Cotton Research, Chinese Academy of Agricultural Sciences, Anyang, China; ^3^ Department of Plant Breeding and Genetics, University of Agriculture, Faisalabad, Pakistan; ^4^ National Key Laboratory of Cotton Bio-breeding and Integrated Utilization, Anyang Institute of Technology, Anyang, China

**Keywords:** DEGs, fiber development, *Gossypium hirsutum*, RNA-Seq, WGCNA

## Abstract

**Introduction:**

Upland cotton (*Gossypium hirsutum*) is the main source of natural fiber in the global textile industry, and thus its fiber quality and yield are important parameters. In this study, comparative transcriptomics was used to analyze differentially expressed genes (DEGs) due to its ability to effectively screen candidate genes during the developmental stages of cotton fiber. However, research using this method is limited, particularly on fiber development. The aim of this study was to uncover the molecular mechanisms underlying the whole period of fiber development and the differences in transcriptional levels.

**Methods:**

Comparative transcriptomes are used to analyze transcriptome data and to screen for differentially expressed genes. STEM and WGCNA were used to screen for key genes involved in fiber development. qRT-PCR was performed to verify gene expression of selected DEGs and hub genes.

**Results:**

Two accessions of upland cotton with extreme phenotypic differences, namely EZ60 and ZR014121, were used to carry out RNA sequencing (RNA-seq) on fiber samples from different fiber development stages. The results identified 704, 376, 141, 269, 761, and 586 genes that were upregulated, and 1,052, 476, 355, 259, 702, and 847 genes that were downregulated at 0, 5, 10, 15, 20, and 25 days post anthesis, respectively. Similar expression patterns of DEGs were monitored using short time-series expression miner (STEM) analysis, and associated pathways of DEGs within profiles were investigated. In addition, weighted gene co-expression network analysis (WGCNA) identified five key modules in fiber development and screened 20 hub genes involved in the development of fibers.

**Discussion:**

Through the annotation of the genes, it was found that the excessive expression of resistance-related genes in the early fiber development stages affects the fiber yield, whereas the sustained expression of cell elongation-related genes is critical for long fibers. This study provides new information that can be used to improve fibers in newly developed upland cotton genotypes.

## 1 Introduction

Cotton is one of the most important economic and cash crops in the world. Due to upland cotton having a high yield, the area in which it is grown comprises 95% of the total global cotton-growing area (Yoo and Wendel, 2014). Determining how to improve the quality of upland cotton fiber while ensuring the yield is an urgent problem that remains to be solved (Kim and Triplett, 2001).

Cotton fiber is an extension of epidermal cells present on cotton seed, which extend from a tiny bump of 10 μm–20 μm to a length of 3 cm–6 cm (Kim and Triplett, 2001). The growth of cotton fiber is divided into four stages: fiber initiation [0–3 days post anthesis (DPA)], elongation (3–20 DPA), secondary wall biosynthesis (20–40 DPA), and maturity (40–50 DPA) (Kim and Triplett, 2001; Haigler et al., 2012). During the fiber initiation stage (i.e., 0–3 DPA), hair-like projections begin to appear on the epidermis of cotton seeds (Qin and Zhu, 2011). This is followed by the fiber elongation stage (3–20 DPA). During this stage, fiber development-related genes are expressed in high amounts and elongated fibers form fiber bundles by twisting (Singh et al., 2009). Close to 20 DPA, fiber elongation gradually stops and fibers enter the stage of secondary wall thickening (20–40 DPA) as the expression of secondary wall synthesis-related genes is upregulated (Hinchliffe et al., 2010). The secondary wall is mainly composed of cellulose, and cellulose synthesis is dependent on cellulose synthase and cellulose synthase-like enzymes (Taylor et al., 2003). During the fiber maturation stage (40–50 DPA), fibroblasts undergo dehydration and form mature fibers (Kim and Triplett, 2001; Haigler et al., 2012). The first two stages primarily affect the number and length of fibers, whereas the third and fourth stages are associated with fiber strength (FS) and fineness (Patel et al., 2020).

In recent years, the success in the sequencing, assembly, and publication of the reference genomes of cotton (diploid and allotetraploid) species has made it possible for subsequent research on the genetic mechanisms of various traits at the genomic level to be carried out (Paterson et al., 2012; Du et al., 2018; Hu et al., 2019). In particular, RNA sequencing (RNA-seq) technology has developed rapidly over the past 10 years and has become an indispensable tool for the analysis of differential gene expression/messenger RNA (mRNA) variable splicing at the transcriptome level (Wang et al., 2009). Comparative transcriptomics is used to analyze differentially expressed genes (DEGs) and has proven to be an effective method for the screening of candidate genes at various growth stages. Numerous studies have employed comparative transcriptome analysis to discover the genes related to high-quality cotton fiber (Applequist et al., 2001; Gilbert et al., 2014; Yoo and Wendel, 2014; Islam et al., 2016; Li et al., 2017b; Li et al., 2017c; Lu et al., 2017; Zou et al., 2019; Jiang et al., 2021a). A comparative analysis of the fiber development transcriptomes of two short-fiber mutants and control cotton samples in different environments identified 88 fiber elongation-related DEGs (Gilbert et al., 2014). The comparative transcriptome analysis of upland cotton MD52ne and MD90ne and their near isogenic lines revealed that FS may be associated with ethylene and its related hormone pathways, in addition to the signaling pathways of receptor-like kinases (RLKs) (Islam et al., 2016). Moreover, the comparative analysis of the MBI9915 and MBI9749 transcriptomes of chromosome segment substitution lines (CSSLs) has provided new insights into the biosynthesis-related pathway genes of the secondary wall (Li et al., 2017b). The application of transcriptome analysis for a population of upland cotton recombinant inbred lines (RILs) identified several candidate genes associated with fiber initiation and FS (Zou et al., 2019; Jiang et al., 2021a; Jiang et al., 2021b). However, studies that have employed a systematic analysis on all stages of fiber development are limited.

In this study, RNA-seq technology was used to conduct a comparative transcriptome analysis of the plant material ZR014121, which demonstrates excellent fiber quality and poor yield, and line EZ60, which demonstrates poor fiber quality and high yield. The primary focus of this study was to report differences in the transcription level of the genes involved in fiber development. Through DEG analysis and weighted gene co-expression network analysis (WGCNA), candidate key genes that may be related to fiber development were identified. These findings can be used in the development of new genetic material with the potential to improve cotton fiber quality and yield.

## 2 Materials and methods

### 2.1 Plant materials

ZR014121, with its excellent fiber quality and poor yield, and EZ60, with its poor fiber quality and high yield, were grown in standard field conditions at the Experimental Station of the Institute of Cotton Research, Chinese Academy of Agricultural Sciences, located in Anyang, Henan, China (36°N, 114°E) in 2020. EZ60 and ZR014121 are preserved in the National Germplasm Library (Anyang, Henan, China) under accession numbers M116025 and ZM115357, respectively. The plant material was sown in rows that were 3 m long and 0.8 m wide. Ten rows of each plant material were planted. Planting and sampling occurred in April and September, respectively. Field management techniques followed those of regular breeding practices. The days to flowering were recorded as DPA, and cotton bolls were marked individually. The cotton bolls were collected at 0, 5, 10, 15, 20, and 25 DPA, and fiber was extracted from each boll with a sterile scalpel and frozen in liquid nitrogen for the subsequent experiments. Sampling from each period was conducted with three biological replicates. The samples for each period for ZR014121 and EZ60 were labeled ZR_0D, ZR_5D, ZR_10D, ZR_15D, ZR_20D, and ZR_25D and EZ_0D, EZ_5D, EZ_10D, EZ_15D, EZ_20D, and EZ_25D, respectively. The data collected pertained to the fiber-quality traits from two cotton accessions planted in two different locations over 2 years (20AnYang, 21AnYang, 20HeBei, and 21HeBei).

### 2.2 RNA isolation, library construction, and RNA-seq analysis

The total RNA of the fiber samples was extracted using the RNAprep Pure Plant Kit (Polysaccharides & Polyphenolics-rich) (Tiangen, Beijing, China), and 1% agarose gel electrophoresis was used for the detection of RNA degradation and contamination. The RNA concentration was determined using a NanoDrop 2000 spectrophotometer (Thermo Science, Waltham, MA, USA). The RNA integrity was evaluated using a RNA Nano 6000 Assay Kit for the Bioanalyzer 2100 system (Agilent Technologies, Palo Alto, CA, USA). Approximately 2 µg RNA per sample was used to construct the transcriptome library using an Illumina TruSeq™ RNA Sample Preparation Kit (Illumina, San Diego, CA, USA). Transcriptome sequencing of 36 libraries was carried out on the Illumina Novaseq 6000 sequencing platform that produced 150 base pair (bp) paired-end (PE) raw reads (BerryGenomics Co., Ltd., Beijing, China). The raw data were generated in FASTQ format and processed using Trimomatic version 0.39 (Bolger et al., 2014). Reads containing linkers and poly-N, low-quality reads with ≥10% unknown nucleotides (N) and >20% bases, and reads with a phred quality score <5 were excluded. The GCpercentage and Q30 of the clean data were then calculated to evaluate the quality for downstream analysis. HISAT2 v2.1.0 was used to build an index of reference genomes with default parameters (Pertea et al., 2016), with the sequence alignment following the *G. hirsutum* genomes (https://www.cottongen.org/) (Hu et al., 2019). StringTie version 1.3.5 was then employed to quantify the fragments per kilobase of exon per million reads (FPKM) values of the genes (Pertea et al., 2015). Pearson correlation analysis was used to evaluate the correlation between samples. Samples with a Pearson correlation coefficient of less than 0.8 among the three biological replicates were deleted from the database.

### 2.3 Differentially expressed gene analysis

The DESeq2 package in R (R Core Team) was used to screen DEGs between the two cotton accessions at a particular fiber developmental stage (vertical) and among stages of the same accession (horizontal) based on the count number of each gene transcript (Love et al., 2014). The DESeq2 package was also employed for principal component analysis (PCA) and the visualization of the results (Love et al., 2014). The screening criteria were as follows: false discovery rate (FDR) <0.05; log_2_Fold-change of pairwise comparison >1 or <−1; and FPKM ≥0.5. Gene annotations for all DEGs were obtained from the Cottongen database (https://www.cottongen.org/) (Yu et al., 2014).

STEM software was used to analyze the expression patterns of DEGs in the two accessions (Ernst et al., 2005). To explore the functions of DEGs, the Kobas3.0 tool was used to perform Kyoto Encyclopedia of Genes and Genomes (KEGG) and gene ontology (GO) analysis based on the clusterProfiler package in R and the Cottongen database (https://www.cottongen.org/) (Altschul et al., 1990; Kanehisa and Goto, 2000; Wu et al., 2006; Xie et al., 2011; Yu et al., 2014; Kanehisa, 2019; Kanehisa et al., 2021; Wu et al., 2021).

### 2.4 Construction of co-expression networks and screening of hub genes

The WGCNA package in R was used to analyze the gene co-expression network and screen the genes related to fiber quality (Langfelder and Horvath, 2008). The edge files generated after co-expression network analysis were sorted according to weight value. The first 200 pairs of network connections were used to establish the co-expression network. In each module, hub genes were screened according to their module membership (K_ME_) values. Cytoscape 3.9.0 (Shannon et al., 2003) was employed to vizualize the co-expression network.

### 2.5 Hub genes and DEGs expression pattern validation

Quantitative real-time PCR (qRT-PCR) was used to verify the gene expression of select DEGs and hub genes. Approximately 1 µg of the total RNA was used as a template to synthesize the cDNA in the HiScript^®^ II Q RT SuperMix for qPCR (+gDNA wiper) (Vazyme Biotech Co., Ltd). Real-time quantitative PCR was carried out using the ChamQTM Universal SYBR^®^ qPCR Master Mix (Vazyme Biotech Co., Ltd) and LightCycler^®^ 480 II Real-time PCR instrument (Roche, Basel, Switzerland). The qPrimerDB website (https://biodb.swu.edu.cn/qprimerdb/) was used as a reference to design qRT-PCR-specific primers (Lu et al., 2018) (**Supplementary Table S8**). The gene expression level was calculated using the 2^−△△CT^ method with three biological replicates (Livak and Schmittgen, 2001).

## 3 Results

### 3.1 Phenotypic data analysis of the two accessions

**Figure 1:**
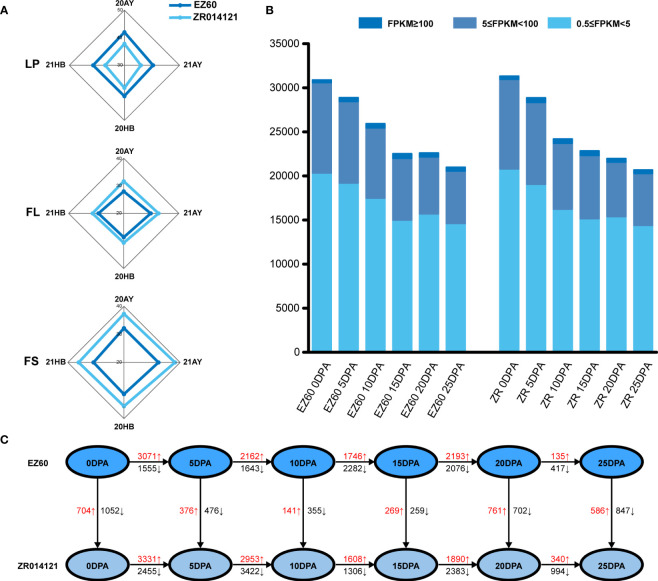
Phenotypic data, transcription level, and DEG statistics for each sample. **(A)** Phenotypic data for LP, FL, and FS of EZ60 and ZR014121 from four environmental conditions. **(B)** Transcript levels at each developmental stage of both accessions statistically classified as 0.5≤FPKM<5, 5≤FPKM<100, and 100≤FPKM. **(C)** The number of DEGs was counted for the same period of both accessions in various developmental periods. The number of upregulated and downregulated genes is marked in red and black. DEGs, differentially expressed genes; FL, fiber length; FPKM, fragments per kilobase of exon model per million mapped fragments; FS, fiber strength; LP, lint percentage.

The lint percentage (LP) is a key measure for the quantification of cotton fiber yield, whereas FS and fiber length (FL) are important fiber-quality traits. The yield and quality potential of selected cited genotypes were recorded in four environments, namely, 20AnYang, 21AnYang, 20HeBei, and 21HeBei. The average values of LP, FL, and FS determined for EZ60 were 40.82%, 28.91 mm, and 31.66 cN/tex, respectively, and those for ZR014121 were 37.32%, 31.39 mm, and 36.8 cN/tex, respectively (**Figure 1A** and **Supplementary Table S1**). Thus, EZ60 has a higher fiber yield and ZR014121 has a higher fiber quality.

### 3.2 Transcriptome sequencing analysis and correlation of replicate samples

Fiber samples were collected during fiber growth (i.e., at 0 DPA, 5 DPA, 10 DPA, 15 DPA, 20 DPA, and 25 DPA) to identify the key genes affecting fiber quality. Transcriptome sequencing was subsequently carried out. A total of 1,634.76 million clean reads were retrieved from 36 libraries, with an average number of reads per sample of 45.41 million. Moreover, 97.01% to 99.36% of Q30 was calculated with an average of 98.80%, whereas 43% to 45% of the GC contents were calculated with an average of 44.17% (**Supplementary Table S2**). Note that two samples were removed as their correlation coefficients were less than 0.8 (**Supplementary Table S3**).

Based on the FPKM value, we believed that genes with an FPKM value greater than 0.5 were expressed in this study. A total of 30,893, 28,885, 25,928, 22,518, 22,613, and 20,992 genes were expressed at six time points (0 DPA, 5 DPA, 10 DPA, 15 DPA, 20 DPA, and 25 DPA, respectively) in EZ60. Similarly, 31,325, 28,857, 24,188, 22,835, 21,973, and 20,683 genes were expressed in ZR014121 (at 0 DPA, 5 DPA, 10 DPA, 15 DPA, 20 DPA, and 25 DPA, respectively). Among all expressed genes, those with FPKM values of 0.5 to 5, 5 to 100, and ≥100 accounted for 67.44%, 30.83%, and 1.73% of the total gene models, respectively (**Figure 1B**).

### 3.3 Analysis of differentially expressed genes

To identify the genes involved in the development of fiber quality, differentially expressed genes were analyzed using the DESeq2 package in R for the different fiber stages of both accessions through vertical and horizontal comparisons. After removing duplicate genes, 19,915 DEGs were identified during cotton fiber development (**Figure 1C** and **Supplementary Tables S4, 5**).

At 0 DPA, when comparing ZR014121 to EZ60, 1,756 DEGs (FPKM ≥0.5 and FDR <0.05) were identified, of which 704 genes were upregulated (log_2_(FC)>1) and 1,052 genes were downregulated (log_2_(FC)<−1). We also screened high-expression genes that exhibited significant differences in their expression (FPKM ≥10, |log_2_(FC)|>2). A total of 34 genes were upregulated and 61 genes were downregulated in the developmental stages (**Supplementary Table S6**). Among the upregulated genes, two genes (*GH_A08G1451* and *GH_D07G1411*) encoding S-adenosylmethionine synthase were responsible for the production of S-adenosylmethionine. *GH_A08G2565* was annotated as the Alpha-1,4-glucan-protein synthase family protein, and participated in the amino sugar and nucleotide sugar metabolism process. In addition, *GH_D01G0810* was enriched in the ascorbate and aldarate metabolism pathway, annotated as Vitamin C Defective 2. Among the downregulated genes, *GH_A10G0118* was annotated as starch synthase 3 (SS3). Two members of the aldehyde dehydrogenase (ALDH) family were identified and annotated as ALDH10A8 (*GH_D11G0455* and *GH_A11G0436*), whereas *GH_D07G1876* was annotated as encodsphosphoserine aminotransferase 2.

At 5 DPA, ZR014121 was compared with EZ60, and 852 DEGs (FPKM≥0.5 and FDR<0.05) were identified, 376 of which were upregulated (log_2_(FC)>1) and 476 of which were downregulated (log_2_(FC)< −1). We also assessed the high-expression genes with significant differences in their expression level (FPKM≥10, |log_2_(FC)|>2). Among them, 36 and 34 genes were upregulated and downregulated, respectively. The majority of upregulated genes were related to proteins involved in various processes of the endoplasmic reticulum. Several genes encoding heat shock proteins were identified, including heat shock cognate protein 70-1 (*GH_D05G0960* and *GH_A05G0973*), heat shock protein 70B (*GH_A13G2624*), heat shock protein 90.1 (*GH_A12G2751*), 17.6kDa class II heat shock proteins (*GH_A07G0216* and *GH_D07G0225*), and mitochondrion-localized small heat shock protein 23.6 (*GH_D12G2202*). In addition, two genes encoding ubiquitin-conjugating enzymes were also upregulated and annotated as ubiquitin-conjugating enzyme 10 (*GH_A08G1154*) and ubiquitin-conjugating enzyme 22 (UBC22) (*GH_D11G3489*). Among the downregulated genes, *GH_A05G4206* was annotated as 10-formyltetrahydrofolate synthetase, and *GH_A08G2085* and *GH_D08G2099* were annotated as beta-6 tubulin.

At 10 DPA, ZR014121 was compared with EZ60, and 496 DEGs (FPKM≥0.5 and FDR<0.05) were identified, of which 141 were upregulated (log_2_(FC)>1) and 355 were downregulated (log_2_(FC)<−1). We also screened the genes for high expression and significant differences in their expression (FPKM≥10, |log_2_(FC)|>2). Among them, 9 genes were upregulated and 37 genes were downregulated. For the upregulated genes, *GH_A08G1447* was annotated as ADP-ribosylation factor A1F. Some genes were also enriched in the ribosomal pathway (*GH_D11G0050*, *GH_D13G2117*, and *GH_D02G0697*). Among the downregulated genes, *GH_D02G1053* was annotated as the phosphofructokinase family protein. Phosphoserine aminotransferase 2 (*GH_D07G1876*) was found to be downregulated at 0 and 10 DPA.

At 15 DPA, ZR014121 was compared with EZ60, and 528 DEGs (FPKM≥0.5 and FDR<0.05) were identified. A total of 269 genes were upregulated (log_2_(FC)>1) and 259 genes were downregulated (log_2_(FC)< −1). We also assessed the genes with a high expression and significant differences in their expression (FPKM≥10, |log_2_(FC)|>2). Among them, 31 genes were upregulated and 34 genes were downregulated. Among the upregulated genes, three genes were enriched for the fatty-acid elongation pathway and annotated as 3-ketoacyl-CoA synthase 6 (*GH_A03G1679* and *GH_D02G1843*) and 3-ketoacyl-CoA synthase 1 (*GH_D12G1341*). *GH_A08G2414* was annotated as O-acetylserine (thiol) lyase (OAS-TL) isoform A1 and encoded cytosolic O-acetylserine (thiol) lyase. Among the downregulated genes, *GH_D06G1226* was annotated as peroxidase 2. Interestingly, the upregulated gene *GH_A08G1447* was present at 10 DPA, yet this gene was downregulated at 15 DPA.

At 20 DPA, ZR014121 was compared with EZ60, and 1,463 DEGs (FPKM≥0.5 and FDR<0.05) were identified, of which 761 genes were upregulated (log_2_(FC)>1) and 702 genes were downregulated (log_2_(FC)<−1). We also screened genes with a high expression and significant differences in their expression (FPKM≥10, |log_2_(FC)|>2). Among them, 48 genes were upregulated and 90 genes were downregulated. Among the upregulated genes, two genes (*GH_D12G2066* and *GH_D09G2122*) were enriched in the pentose and glucuronate interconversions pathway and annotated as the pectate lyase family protein and pectin lyase-like superfamily protein, respectively. Genes enriched in metabolic pathways were also identified, including 3-ketoacyl-CoA synthase 6 (*GH_D01G2106*, *GH_A01G2012*, and *GH_D02G1843*), 3-ketoacyl-CoA synthase 10 (*GH_D13G2158*), alcohol dehydrogenase 1 (*GH_A01G2058*), and D-3-phosphoglycerate dehydrogenase (*GH_D12G0221*). *GH_A03G0664* encoded the ferulyl-CoA transferase. Among the downregulated genes, the glycerolipid metabolism pathway was enriched, including glycerol-3-phosphate acyltransferase 5 (*GH_A10G2449*, *GH_D04G0663*, and *GH_D10G2557*) and glycerol-3-phosphate acyltransferase 8 (*GH_A11G0931*). In phenylpropanoid biosynthesis, *GH_D10G0525* and *GH_A05G0051* were annotated as 4-coumaric acid: CoA ligase 1 and 4-coumaric acid: CoA ligase 2, respectively. Asparaginase B1 (*GH_D02G0918*) was enriched in the biosynthesis of secondary metabolites. In addition, two peroxidase super-family proteins (*GH_A10G1988* and *GH_D10G2089*) were overexpressed in EZ60 as compared with ZR014121.

At 25 DPA, ZR014121 was compared with EZ60, and 1,433 DEGs (FPKM≥0.5 and FDR<0.05) were identified, where 586 genes were upregulated (log_2_(FC)>1) and 847 genes were downregulated (log_2_(FC)< −1). We also screened genes with a high expression and significant differences in their expression (FPKM≥10, |log_2_(FC)|>2). Among them, 34 genes were upregulated and 61 genes were downregulated. Among the upregulated genes, *GH_D11G0356* was enriched in the carbon metabolism pathway and was annotated as malate dehydrogenase. Interestingly, *GH_A03G0664* was upregulated at 20 DPA and 25 DPA. Among the downregulated genes, *GH_A09G0134* was annotated as pectin methylesterase 31. Note that *GH_D02G0918* was not only downregulated at 20 DPA, but the expression level in EZ60 was higher than normal.

### 3.4 Principal component analysis

**Figure 2:**
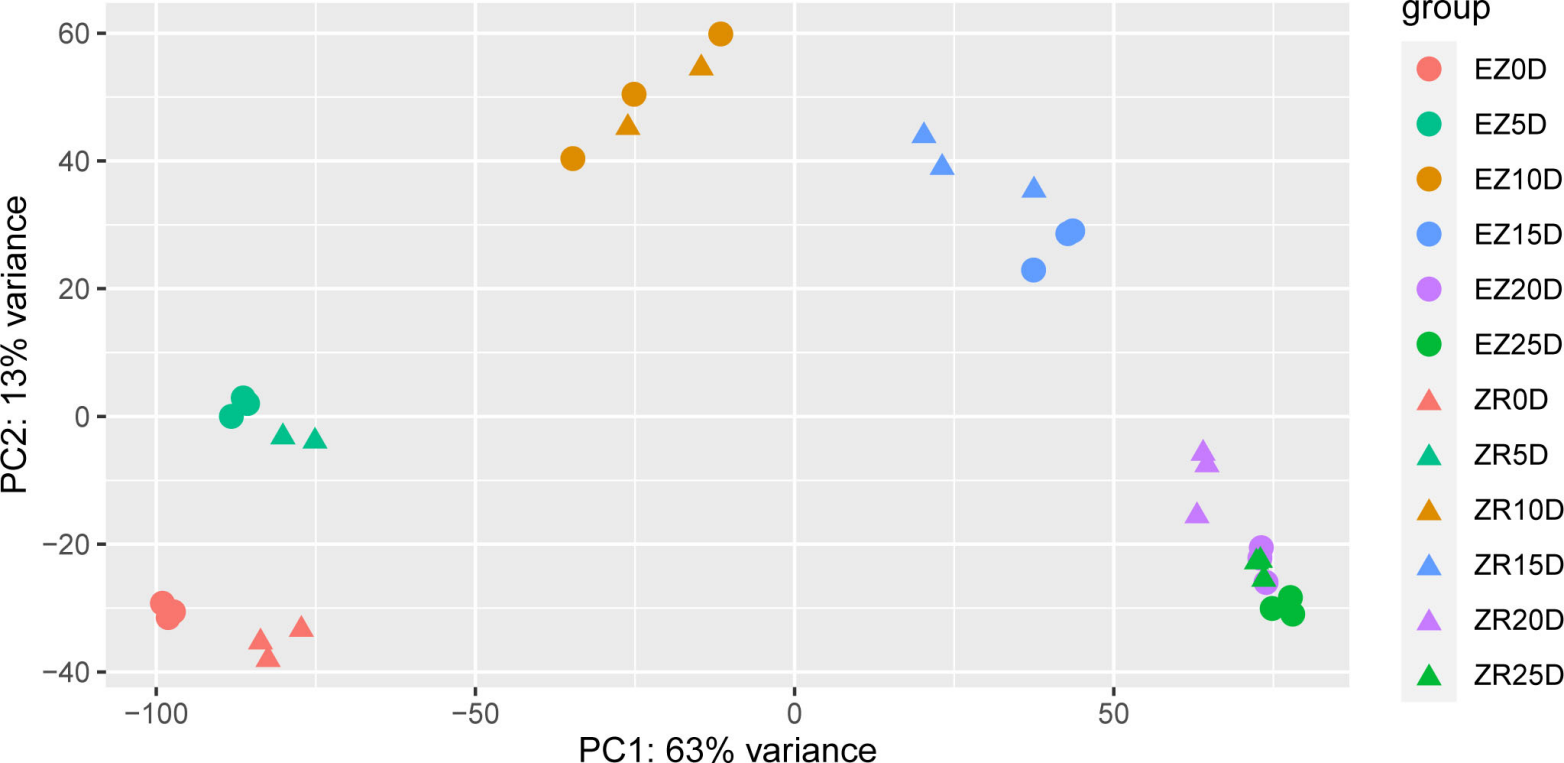
PCA of the two cotton accessions at various time points presented as DPAs. The same color represents a single timeline, and the circles and triangles represent EZ60 and ZR014121, respectively. DPAs, days post anthesis; PCA, principal component analysis.

Principal component analysis was carried out during six periods of fiber development in the two cotton accessions so that the periods at which the two accessions exhibited differences in fiber development could be determined (**Figure 2**). Among them, there were significant differences in the transcript levels between the two accessions at 0 DPA, 5 DPA, 15 DPA, and 20 DPA. Note that ZR 25D and EZ 20D were almost identical in terms of the sample variability.

### 3.5 Temporal gene expression patterns analysis

**Figure 3:**
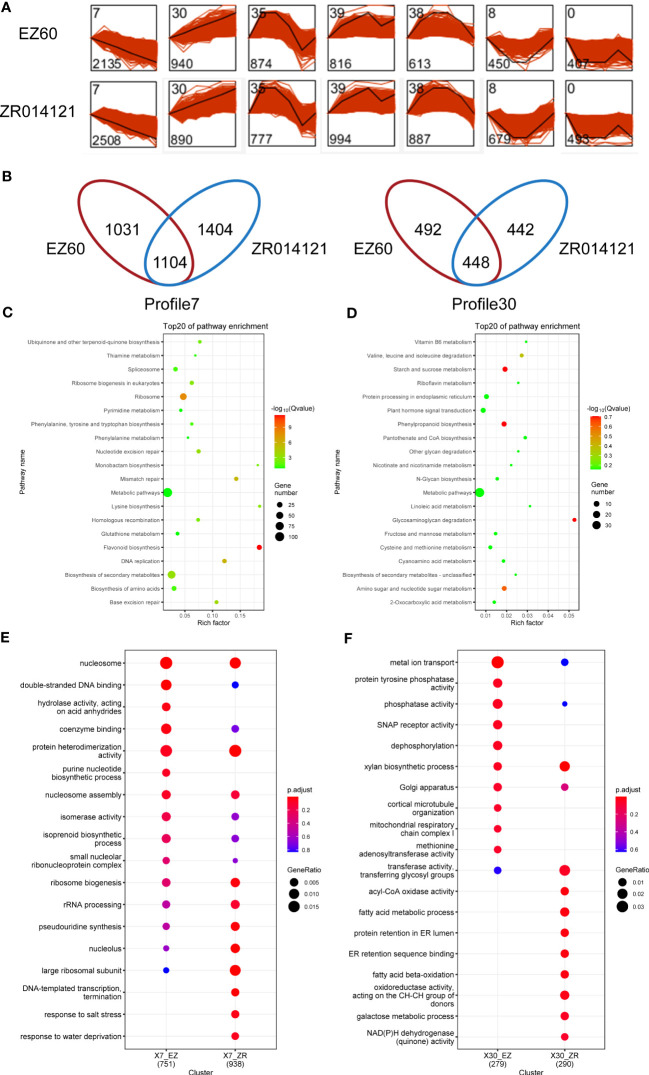
Description of DEG analysis in upland cotton. **(A)** STEM analysis results, bottom left corner of each profile is gene number and top left corner is profile ID. **(B)** Venn diagram visualizing the gene number in profile7 and profile30 for both accessions. **(C)** KEGG analysis of the same genes in the two accessions in profile7. **(D)** KEGG analysis of the same genes in the two accessions in profile30. **(E)** GO analysis of different genes in the two accessions in profile7. **(F)** GO analysis of different genes in the two accessions in profile30. DEGs, differentially expressed genes; GO, gene ontology; KEGG, Kyoto Encyclopedia of Genes and Genomes; STEM, short time-series expression miner.

To analyze the temporal expression, we carried out STEM analysis on all DEGs in EZ60 and ZR014121. In particular, the 12,786 and 14,563 DEGs in EZ60 and ZR014121, respectively, were divided into seven profiles (**Figure 3A**). The genes in each profile exhibited similar expression patterns. To investigate the genes associated with fiber development, we focused on profiles (profile7 and profile30) that were consistently upregulated or downregulated with fiber development. Profile7 contained 2,135 genes for EZ60 and 2,508 genes for ZR014121, and 1,104 genes were found to be identical. Profile30 contained 940 genes for EZ60 and 890 genes for ZR014121, and 448 were found to be identical. In profile7, 1,031 and 1,404 genes were specifically expressed in EZ60 and ZR014121, whereas in profile30, 492 and 442 genes were specifically expressed in EZ60 and ZR014121, respectively (**Figure 3B**).

To investigate the pathways with common expression patterns in two accessions, we carried out KEGG enrichment analysis of the genes common to both accessions (**Supplementary Table S7**). The DEGs in profile7 were primarily enriched in metabolic pathways, secondary metabolite biosynthesis, ribosome, and flavonoid biosynthesis (**Figure 3C**). In contrast, DEGs in profile30 were enriched in the phenylpropanoid biosynthesis, starch and sucrose metabolism, glycosaminoglycan degradation, and amino sugar and nucleotide sugar metabolism (**Figure 3D**).

GO enrichment analysis with specific DEGs was conducted to explore the differences between the two accessions (**Supplementary Table S7**). In profile7, EZ60 was compared with ZR014121 based on the Venn diagram in **Figure 3E**. The specific DEGs of EZ60 were enriched for the GO terms of GO:0016817 “hydrolase activity, acting on acid anhydrides,” and GO:0006164 “purine nucleotide biosynthetic process”. Moreover, GO:0006353 “DNA-templated transcription, termination”, GO:0009651 “response to salt stress”, and GO:0009414 “response to water deprivation” were specifically enriched in the DEGs of ZR014121. In profile30, ZR014121 was compared with EZ60, as shown in the Venn diagram in **Figure 3F**. The specific DEGs of ZR014121 were enriched to the GO terms of GO:0003997 “acyl-CoA oxidase activity”, GO:0006631 “fatty acid metabolic process”, GO:0006621 “protein retention in ER lumen”, GO:0046923 “ER retention sequence binding”, GO:0006635 “fatty acid beta-oxidation”, GO:0016627 “oxidoreductase activity, acting on the CH-CH group of donors”, GO:0006012 “galactose metabolic process”, and GO:0003955 “NAD(P)H dehydrogenase (quinone) activity”. The GO enrichment results indicate that there are large differences between the two accessions in terms of the enriched GO terms, which may affect the development of fiber.

### 3.6 Gene co-expression network analysis and identification of hub genes in correlation networks

We constructed a co-expression network of 5,414 DEGs using WGCNA to investigate the relationship between gene expression and fiber development and identify the genes associated with fiber development.

**Figure 4:**
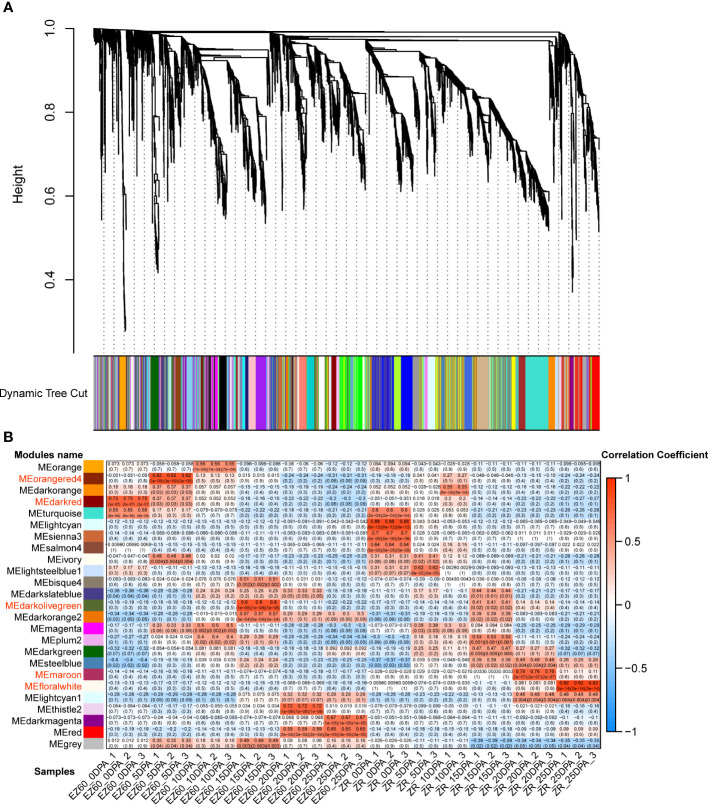
Gene co-expression network analysis of vertical DEGs for the two cotton accessions. **(A)** Gene dendrogram of the co-expression modules in WGCNA. According to the expression pattern, 5,414 DEGs were dynamically cut into different-colored modules. **(B)** Correlation analysis of 25 co-expression modules with 34 samples; important modules are marked in red. DEGs, differentially expressed genes; WCGNA, weighted gene co-expression network analysis.

The hierarchical clustering method was used to construct the topological overlap matrix, merging the dynamic cut modules with similar expression patterns. A total of 25 modules were identified in the fiber samples of both accessions. Five of these (i.e., darkred, orangered4, darkolivegreen, maroon, and floralwhite) were highly correlated with each period of fiber development (**Figure 4**). In five significant modules, a total of 20 DEGs were identified as hub genes based on the highest K_ME_ values in each module. All hub genes exhibited K_ME_ values greater than 0.9 (**Table 1**). The gene co-expression networks for the five significant modules are provided in the additional files (**Supplementary Figures S1–S5**).

**Table 1:** Candidate hub genes in five modules.

Gene ID	K_ME_	*Arabidopsis* ID	Description
darkred module (EZ_0DPA)			
*GH_A12G0442*	0.971	*AT1G30460*	Cleavage and polyadenylation specificity factor 30
*GH_D08G0448*	0.967	*AT1G09680*	PPR superfamily protein
*GH_D10G0843*	0.962	*AT1G80070*	Pre-mRNA-processing-splicing factor
*GH_A08G1344*	0.958	*AT1G64090*	Reticulan-like protein B3
*GH_D08G0264*	0.958	*AT3G20630*	Ubiquitin-specific protease 14
*GH_A10G0118*	0.957	*AT1G11720*	SS3
*GH_D11G0143*	0.956	*AT3G49240*	PPR superfamily protein
*GH_A02G1327*	0.951	*AT5G04170*	Calcium-binding EF-hand family protein
orangered4 module (EZ_5DPA)			
*GH_D03G1709*	0.953	*AT5G23860*	Tubulin beta 8
*GH_A04G1196*	0.945	*AT5G08680*	ATP synthase alpha/beta family protein
darkolivegreen module (EZ_15DPA)			
*GH_D08G2099*	0.971	*AT5G12250*	Beta-6 tubulin
*GH_A05G3885*	0.961	*AT3G01120*	PLP-dependent transferases superfamily protein
*GH_A08G2085*	0.959	*AT5G12250*	Beta-6 tubulin
*GH_A08G0145*	0.954	*AT4G08810*	Calcium ion binding
*GH_D11G1234*	0.954	*AT4G15800*	RALF-like 33
maroon module (ZR_20DPA)			
*GH_A01G2058*	0.945	*AT1G77120*	Alcohol dehydrogenase 1
*GH_D08G0287*	0.924	*AT5G57560*	Xyloglucan endotransglucosylase/hydrolase family protein
*GH_A05G2091*	0.919	*AT5G67360*	Subtilase family protein
floralwhite module (ZR_25DPA)			
*GH_D05G3615*	0.943	*AT1G29140*	Pollen Ole e 1 allergen and extensin family protein
*GH_D08G2716*	0.938	*AT2G25490*	EIN3-binding F box protein 1

K_ME_, eigengene connectivity value; mRNA, messenger RNA; PLP, pyridoxal phosphate; PPR, pentatricopeptide repeat; SS3, starch synthase 3.

Eight hub genes were identified in the darkred module (related to EZ60_0DPA), including the pre-mRNA-processing-splicing factor, reticulan-like protein B3, ubiquitin-specific protease 14, SS3, calcium-binding EF-hand family protein, and cleavage and polyadenylation specificity factor 30. In the orangered4 module (related to EZ60_5DPA), two genes were identified as hub genes, namely, tubulin beta 8 and an ATP synthase alpha/beta family protein. Five genes were identified as hub genes in the darkolivegreen module (related to EZ60_15DPA), namely, beta-6 tubulin, pyridoxal phosphate (PLP)-dependent transferases superfamily protein, calcium ion binding, and rapid alkalization factor (RALF)-like 33. In the maroon module (related to ZR_20DPA), three genes were identified as hub genes, namely, alcohol dehydrogenase 1, xyloglucan endotransglucosylase/hydrolase family protein, and subtilase family protein. Two genes were identified as hub genes in the floralwhite module (related to ZR_25DPA), namely, the pollen Ole e 1 allergen and extensin family protein and EIN3-binding F box protein 1. These may be key regulatory genes in fiber development.

### 3.7 Validation of qRT-PCR expression pattern

**Figure 5:**
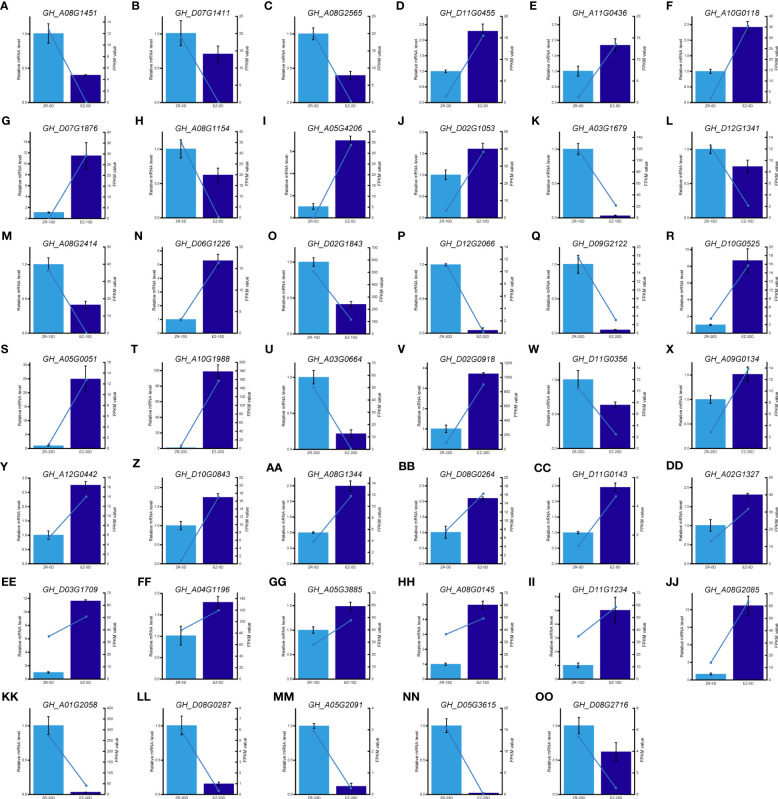
qPCR analysis of hub genes and key DEGs. **(A–X)** qPCR results of key DEGs during fiber development. **(Y–OO)** qPCR results of hub genes in five key modules. DEGs, differentially expressed genes; qPCR, quantitative PCR.

qRT-PCR was carried out to verify the expression pattern of important DEGs and hub genes. The expression of 41 genes was monitored, indicating that the significant results were similar to the FPKM values in the transcriptome analysis (**Figure 5**). These results validated the reliability of our RNA-seq.

## 4 Discussion

### 4.1 Insights into the RNA-seq of divergent accessions

RNA-seq was conducted on two accessions with extreme phenotypic differences, EZ60 (LP of 40.82%, FL of 28.91 mm, and FS of 31.66 cN/tex) and ZR014121 (LP of 37.32%, FL of 31.39 mm, and FS of 36.80 cN/tex) to explore the molecular mechanisms involved in the fiber development of cotton. The recent application of RNA-seq techniques to study fiber development generally focuses on specific timelines and periods, but research explaining the whole fiber development process is lacking. Based on the analysis of two short-fiber mutants and the fiber transcriptome of wild type (WT) grown in different environments, Gilbert et al. (2014) identified 88 differentially expressed genes required for fiber elongation. Li et al. (2017b) provided new insights into the biosynthesis-related pathways of secondary walls in chromosome segment substitution lines (CSSLs) by comparative analysis of the MBI9915 and MBI9749 transcriptomes in CSSLs. Similarly, Zou et al. (2019) and (Jiang et al. 2021a, Jiang et al., 2021b) identified several candidate genes related to FS and fiber initiation, respectively, using the transcriptome analysis of a recombinant inbred lines (RILs) population of upland cotton.

RNA-seq was carried out on cotton fibers from 0–25 days after flowering to generate valuable data that could explain the genetic and molecular mechanisms of fiber development. Such data sets play an integral role in breeding work. In this study, 1.635 billion clean reads were obtained in 36 libraries, with an average of 45.41 million reads per sample. The average Q30 and GC contents were determined to be 98.80% and 44.17%, respectively. This demonstrates the reliability of the presented RNA-seq data. Data with a low correlation (<0.8) were removed due to possible environmental influences on the samples. These data act as a basis for the exploration of differences in transcript levels during fiber development.

### 4.2 DEG analysis reveals differences in fiber development processes

Differentially expressed gene analysis was carried out to explore the differences in transcript levels during fiber development. At 0 DPA, *GH_A10G0118* (**Figure 5F**), annotated as SS3, was significantly upregulated in the expression of EZ60. SS3 has been reported to promote the release of glucose units by modifying the cell wall structure (Grisolia et al., 2017; Gámez-Arjona and Mérida, 2021). This process leads to the metabolism of energy and the accumulation of sugars. In particular, it may provide energy for the differentiation of seed epidermal cells. For the high fiber-yielding accession EZ60, during this period DEGs are enriched in metabolic pathways such as glycine, serine, and threonine metabolism. In addition, a variety of amino acid synthesis-related enzymes have high FPKM values in EZ60. For example, phosphoserine aminotransferase 2 (*GH_D07G1876*) was regulated and expressed by MYB34, playing an important role in plant development and metabolism (Benstein et al., 2013). The ALDH10A (*GH_D11G0455* and *GH_A11G0436*) enzyme participates in seed development by synthesizing γ-butyrobetaine (Jacques et al., 2020; Tola et al., 2020). This implies that a large number of amino acids is synthesized and accumulated during the differentiation of seed epidermal cells, a process that may affect the quantity of cotton fibers produced. At 5 DPA, two beta-6 tubulin genes (*GH_A08G2085* and *GH_D08G2099*) (**Figure 5JJ**) were significantly upregulated in EZ60. These genes are involved in the direction of cell growth by directing fibrillin (Soga et al., 2018) and may be required to regulate the growth of fibers during the early stages of fiber elongation. The synthesis of protein and various metabolism processes are more active during fiber elongation. 10-formyltetrahydrofolate synthase (*GH_A05G4206*) can be transported as mRNA to distant cells, regulating cell differentiation and growth (Thieme et al., 2015). This provides the impetus for fiber elongation. During the elongation stage of fiber development (10 DPA and 15 DPA), *GH_A08G1447*, annotated as ADP-ribosylation factor A1F, was significantly upregulated in the expression of the high fiber quality of ZR014121. This gene is involved in the transport process of vesicles by assisting in transporting cellulose synthase to the plasma membrane and non-cellulose polysaccharides to the cell wall (Gebbie et al., 2005). This implies that *GH_A08G1447* plays an important role in cell wall composition and in biosynthesis during fiber elongation stage. In addition, three genes (*GH_A03G1679*, *GH_D02G1843*, and *GH_D12G1341*) (**Figure 5K**) are upregulated in the fiber elongation of ZR014121. They are enriched in the fatty-acid elongation pathway, which regulates the biosynthesis of very long-chain fatty acids (VLCFAs) (Singh et al., 2020; Guyomarc’h et al., 2021). For extracellular lipids, VLCFAs can be classified as either suberin or cuticle/cutin (Li-Beisson et al., 2013). They are essential for the polar transport of growth hormones during plant development and facilitate the process of fiber elongation (Roudier et al., 2010). VLCFA content and the ratio of VLCFAs to short-chain fatty acids have been reported to play a role in the increased production of long fibers (Hu et al., 2020). Moreover, cytosolic O-acetylserine (thiol) lyase can reduce the concentration of cell sulfide to support the structure of actin cytoskeleton (Li et al., 2018). The upregulated expression of these genes provides the foundation for high-quality fiber in ZR014121. The analysis of DEGs revealed the fiber elongation process to be related to metabolic pathways, the biosynthesis of secondary metabolites, and other pathways. Numerous protein transport genes and polar growth-related genes play an important role in this process (Ruan et al., 2001; Qin and Zhu, 2011).

Several DEGs in ZR014121 are upregulated during pre-fiber development and are also associated with resistance. *GH_D01G0810* was significantly upregulated at 0 DPA for ZR014121 and is involved in the ascorbate pathway (Iwagami et al., 2022). Upregulated genes at 5 DPA were enriched for multiple heat shock proteins involved in stress responses (Dorn et al., 2010; Li et al., 2017a; Cha et al., 2020; Zhao et al., 2021). Moreover, UBC22 (*GH_D11G3489*) is reported to have multiple functions during plant development and stress responses (Wang et al., 2020). Cotton fibers are developed from the epidermal cells of the seeds (Kim and Triplett, 2001). Seed epidermal hairs form a plant defense system to protect seeds, and thus resistance-related genes are significantly expressed in the early development of fiber. However, from a macroscopic perspective, this may take up genetic resources for the differentiation of fiber during early development. ZR014121 was heavily upregulated in resistance-related genes early in fiber development, which may explain its low fiber yield. Genetic resources related to stress response pathways are allocated for fiber development, resulting in a higher biomass (Yoo and Wendel, 2014). The fiber initiation stage is important in determining fiber yield, and significant differences between EZ60 and ZR014121 at 0 DPA may induce differences in yield (**Figure 2**).

During the secondary wall thickening stage (20 DPA and 25 DPA), DEGs were enriched in phenylpropanoid biosynthesis, starch and sucrose metabolism, glycosaminoglycan degradation, and amino acid sugar and nucleotide sugar metabolism pathways. Two pectin lyase family genes (*GH_D12G2066* and *GH_D09G2122*) (**Figure 5P**) were significantly upregulated in terms of their expression in ZR014121 and could induce cell-wall loosening, remodeling, and rearrangement (Sun et al., 2018). *GH_A03G0664* (**Figure 5U**), which encodes feruloyl-CoA transferase, was upregulated at both 20 DPA and 25 DPA in ZR014121. It is involved in several synthesis pathways and regulates the cell wall composition (Gou et al., 2009; Molina et al., 2009). Interestingly, at 20–25 DPA, the majority of upregulated DEGs expressed in EZ60 were enriched in the phenylpropanoid biosynthetic pathway and the thickening of the secondary wall. For example, at 20 DPA, the upregulated glycerol-3-phosphate acyltransferase (*GH_A10G2449*, *GH_D04G0663*, *GH_D10G2557*, and *GH_A11G0931*) is reported to be involved in the biosynthesis of cork polyester (Jayawardhane et al., 2018) and 4-Cumaric acid. Furthermore, CoA ligase (*GH_D10G0525* and *GH_A05G0051*) is involved in the biosynthesis of lignin and the process of secondary wall deposition in cotton fibers (Vanholme et al., 2012; Sun et al., 2015). Peroxidase superfamily proteins (*GH_A10G1988* and *GH_D10G2089*) play a role in the lignification stage of the secondary wall (Hoffmann et al., 2020). Moreover, pectin methylesterase 31 was upregulated at 25 DPA in EZ60, and participated in the pectin remodeling process, possibly affecting the thickening of the fiber secondary wall (Liu et al., 2013; Li et al., 2016). Furthermore, malate dehydrogenase was upregulated at 25 DPA in ZR014121 and may influence fiber elongation during fiber development (Imran et al., 2016). Malate, a product of malate dehydrogenase, drives fiber elongation by enhancing turgor pressure (Dhindsa et al., 1975). This may be due to the longer cell elongation of ZR014121 and the shorter duration of fiber growth in EZ60, which is one of the factors influencing poor-quality fiber. The PCA results demonstrate the similarity between fibers from EZ60 at 20 DPA and those from ZR014121 at 25 DPA in terms of the transcript level (**Figure 2**).

### 4.3 Hub genes identified by WGCNA

WGCNA identified numerous modules associated with each period of fiber development and the hub genes for each module. We conducted an extended study for some modules that exhibited a strong correlation with EZ60 early-fiber and ZR014121 late-fiber samples. These modules were correlated with periods that demonstrated significant differences between the two accessions in the PCA results (**Figure 2**). Among the three modules associated with EZ60, the identified hub genes were highly correlated with seed epidermal hair cell differentiation and elongation initiation. These genes include several transcription factors (cleavage and polyadenylation specificity factor 30 and pre-mRNA-processing-splicing factor), genes which regulate the protein synthesis process (reticulan-like protein B3, calcium-binding EF-hand family protein, and calcium ion binding), and genes involved in the energy supply process (ATP synthase alpha/beta family protein). Cleavage and polyadenylation specificity factor 30 is associated with the metabolic process of mRNA and mediates mRNA N6-methyladenosine (m6A) modifications (Hou et al., 2021). This pre-mRNA-processing-splicing factor affects pre-mRNA shearing and is essential for embryonic development in *Arabidopsis* (Sasaki et al., 2015). This gene may affect the differentiation process of epidermal cells. Reticulan-like protein B3 plays an important role in endoplasmic reticulum modeling (Kriechbaumer et al., 2015). The expression of calcium-binding EF-hand family protein is consistent with the involvement of Ca^2+^ in the regulation changes of cell development (Day et al., 2002). ATP synthase alpha/beta family protein drives the synthesis of mitochondrial ATP; its mRNA can move between cells and is transported to more distant tissues (Thieme et al., 2015). These genes may influence the accumulation of protein synthesis during the differentiation of fiber development initiation and the energy transfer process. In addition, RALF-like 33 is a small peptide that causes rapid extracellular alkalinization and acts as an extracellular signal to regulate plant development (Sharma et al., 2016). Ubiquitin-specific protease 14 is required for the lateral root development in *Arabidopsis* and may be associated with the initiation of fiber development (Majumdar et al., 2021). Note that SS3 and multiple tubulin proteins (tubulin beta 8 and beta-6 tubulin) were identified as hub genes, which is consistent with the results of the DEGs analysis. Thus, the role of the aforementioned genes in the early stages of fiber development is crucial. Previous transcriptome research on the Ligon lintless-1 mutant has reported the importance of starch synthase and tubulin for fiber development (Ding et al., 2014).

Among the two modules associated with ZR014121, the xyloglucan endotransglucosylase/hydrolase family protein acts as a cell wall-modifying enzyme to regulate plant growth by maintaining cell wall homeostasis (Zhang et al., 2022). In addition, EIN3-binding F box protein 1 is involved in ethylene signaling (Vaseva et al., 2018). Previous research has linked fiber strength in cotton to the ethylene signaling pathway (Islam et al., 2016). During fiber development, hub genes play an important role in all developmental stages and influence fiber-yield and -quality traits. Therefore, the functions of these hub genes require further investigation.

## 5 Conclusions

Differentially expressed genes were identified by the transcriptome sequencing of fibers from two cotton accessions (EZ60 and ZR014121) with extreme phenotypic differences at various developmental stages. DEGs with similar expression patterns were classified using STEM analysis, focusing on profiles with persistent upregulated and downregulated expression (profile7 and profile30). Their differences in pathways and classes of action were highlighted by KEGG and GO analysis. DEGs and hub genes associated with each fiber developmental stage were selected by longitudinal comparison and multiple screening, including fiber initiation-related genes (SS3 and tubulin). These genes may be associated with fiber yield. Genes associated with fiber elongation (ADP-ribosylation factor A1F, pectin lyase, and fatty acid extension pathway-related genes) and fiber secondary-wall thickening (feruloyl-CoA transferase, and EIN3-binding F box protein 1-related genes) may be crucial to affecting fiber quality. Our results provide new insights into the molecular mechanisms underlying cotton fiber development.

## Data availability statement

The raw reads for the transcriptomic analysis have been uploaded on the SRA database from NCBI under the BioProject PRJNA821165 (https://www.ncbi.nlm.nih.gov/bioproject/PRJNA821165) and will be available after publication. All data generated or analyzed during this study are included in this published article and its **Supplementary Information Files**.

## Ethics statement

The experiments on plant material in this study comply with relevant institutional, national, and international guidelines and legislation.

## Author contributions

QG, YY, and HS initiated the research. JH and ZX designed the experiments. JH, XJ, PL, and ZZ carried out the transcriptional analysis. JH, ZX, XJ, and SF conducted the DEG analyses and WGCNA. JG grew the plant material for experiments. JH and QG drafted the manuscript. YY, MA, and HS finalized the manuscript. All authors contributed to the interpretation of results and approved the final manuscript.
