# Supplementary material for: Comparative transcriptional and co-expression network analysis of two upland cotton accessions with extreme phenotypic differences reveals molecular mechanisms of fiber development
Source: Front Plant Sci. 2023 Aug 31;14:1189490. doi: 10.3389/fpls.2023.1189490 (PMC10502173; doi:10.3389/fpls.2023.1189490)
Supplement: Supplementary file 1 [file DataSheet_1.pdf]

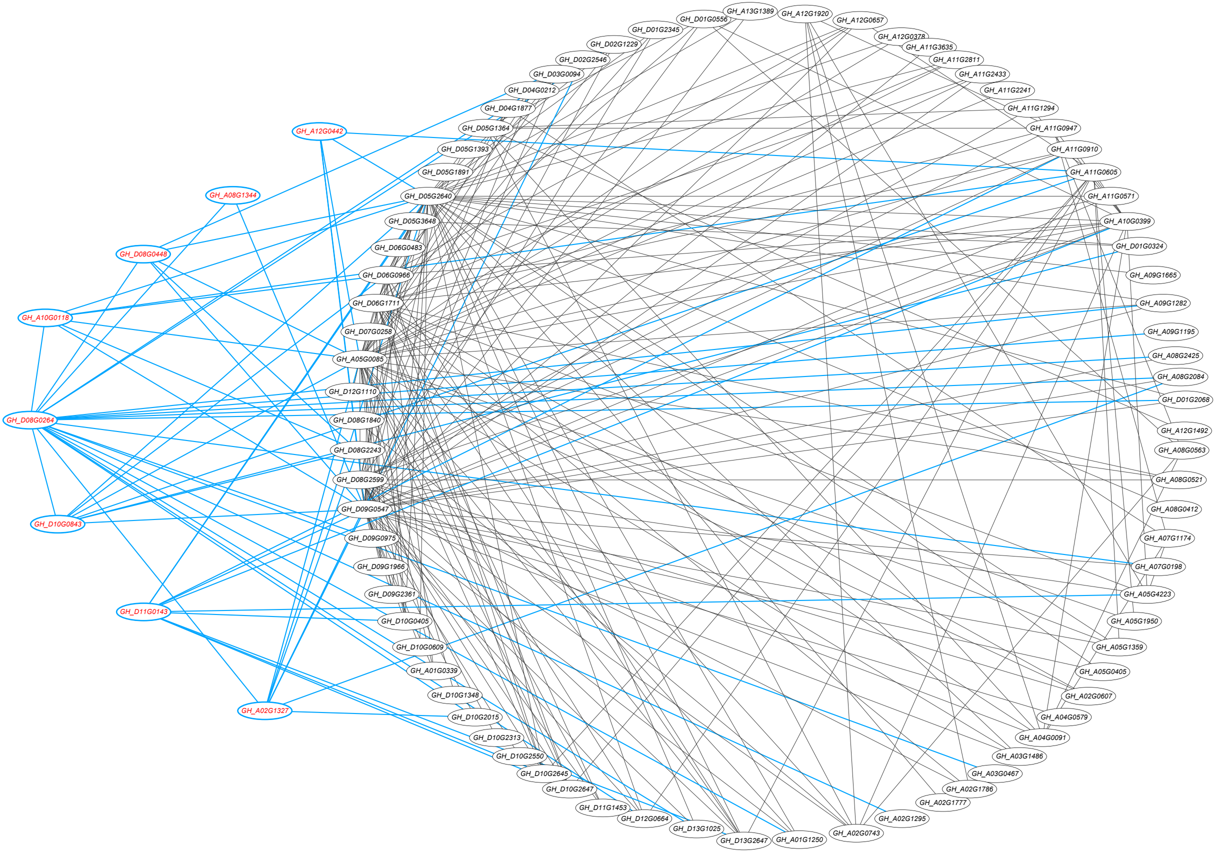

Figure S1 Co-expression network of darkred module (EZ_0DPA).
Hub genes are labeled in red and their co-expression networks are labeled with blue lines.
